# Health-related quality of life loss associated with first-time stroke

**DOI:** 10.1371/journal.pone.0211493

**Published:** 2019-01-28

**Authors:** Yen Shing Yeoh, Gerald Choon-Huat Koh, Chuen Seng Tan, Tian Ming Tu, Rajinder Singh, Hui Meng Chang, Deidre A. De Silva, Yee Sien Ng, Yan Hoon Ang, Philip Yap, Effie Chew, Reshma A. Merchant, Tseng Tsai Yeo, Ning Chou, N. Venketasubramanian, Kim En Lee, Sherry H. Young, Helen Hoenig, David Bruce Matchar, Nan Luo

**Affiliations:** 1 Saw Swee Hock School of Public Health, National University of Singapore, Singapore; 2 Department of Neurology, National Neuroscience Institute, Singapore, Singapore; 3 Department of Neurology, Singapore General Hospital campus, National Neuroscience Institute, Singapore, Singapore; 4 Department of Rehabilitation Medicine, Singapore General Hospital, Singapore, Singapore; 5 Department of Geriatric Medicine, Khoo Teck Puat Hospital, Singapore, Singapore; 6 Department of Rehabilitation Medicine, National University Hospital, Singapore, Singapore; 7 Department of Medicine, Yong Loo Lin School of Medicine, National University of Singapore, Singapore, Singapore; 8 Department of Neurosurgery, National University Hospital, Singapore, Singapore; 9 Raffles Neuroscience Centre, Raffles Hospital, Singapore, Singapore; 10 Farrer Park Medical Centre, Farrer Park Hospital, Singapore, Singapore; 11 Department of Rehabilitation Medicine, Changi General Hospital, Singapore, Singapore; 12 Physical Medicine & Rehabilitation Service, Durham Veterans Affairs Medical Center, Durham, North Carolina, United States of America; 13 Department of Medicine, Duke University School of Medicine, Durham, North Carolina, United States of America; 14 Health Services & Systems Research Programme, Duke-NUS Medical School, Singapore, Singapore; 15 Center for Clinical Health Policy Research, Duke University Medical Center, Durham, North Carolina, United States of America; Qazvin University of Medical Sciences, ISLAMIC REPUBLIC OF IRAN

## Abstract

**Objectives:**

This study aimed to quantify health-related quality of life (HRQoL) loss associated with first episode of stroke by comparing patient-reported HRQoL before and after stroke onset. The impact of stroke in local population was also evaluated by comparing the pre- and post-stroke HRQoL with that of the general population.

**Methods:**

The HRQoL of stroke survivors was assessed with the EQ-5D-3L index score at recruitment, for recalled pre-stroke HRQoL, and at 3 and 12 month post-stroke. Change in HRQoL from pre-stroke to 3 and 12 month was self-reported by 285 and 238 patients, respectively. Mean EQ index score at each time point (baseline: 464 patients; 3 month post-stroke: 306 patients; 12 month post-stroke: 258 patients) was compared with published population norms for EQ-5D-3L.

**Results:**

There was a significant decrease in HRQoL at 3 (0.25) and 12 month (0.09) post-stroke when compared to the retrospectively recalled patients’ mean pre-stroke HRQoL level (0.87). The reduction at 3 month was associated with the reduction in all EQ-5D-3L health dimensions; reductions remaining at 12 month were limited to dimensions of mobility, self-care, usual activities, and anxiety/depression. Stroke patients had a lower mean EQ index than the general population by 0.07 points pre-stroke (0.87 vs. 0.94), 0.33 points at 3 month (0.61 vs. 0.94) and 0.18 points at 12 month (0.76 vs. 0.94) post-stroke.

**Conclusions:**

Stroke has a substantial impact on HRQoL in Singapore, especially in the first three months post-stroke. Compared to the general population, stroke survivors have lower HRQoL even before stroke onset. This pre-stroke deficit in HRQoL should be taken into account when quantifying health burden of stroke or setting goals for stroke rehabilitation.

## Introduction

Prior to the seventies, the health outcomes of disease were primarily measured with the rather limited “hard” end-points, for example, survival and treatment toxicity. Over the past few decades, concern for psychosocial needs of patients has increased dramatically. There is growing awareness that in certain diseases, particularly chronic diseases, health-related quality of life (HRQoL) may be the most important health outcome to consider in revealing the impact of disease [1].

Stroke is one of the most devastating neurological conditions that contributes significantly to the increasing disease burden and death worldwide. Annually, 15 million people worldwide suffer from a stroke, in which 5 million are left permanently disabled, placing a huge burden on family and society. In 2012, stroke was the third leading cause of disability-adjusted life years (DALYs) lost worldwide, which accounted for 1998 DALYs per 100,000 populations. From 1990 to 2010, DALYs had increased by 19% in the Global Burden in Disease Study [2]. Recovery from a stroke is an arduous journey which takes months or years. Many stroke survivors may even never fully recover. It was estimated that the prevalence of stroke survivors with incomplete recovery was 460 per 100,000 and about 30% of these survivors required assistance in at least one activity of daily living [3].

One can prospectively measure individuals’ HRQoL before and after they develop a disease and then use the change in HRQoL to assess disease burden. However, it is sometimes difficult to capture baseline or pre-onset HRQoL of acute-onset conditions such as stroke; stroke occurs in a sudden and unexpected way and participants are usually recruited after the event has occurred. In many studies, HRQoL burden of stroke has been estimated through cross-sectional comparison of persons after stroke and persons without a history of stroke [4, 5] or more commonly, by comparing to population norms generated from HRQoL surveys of the general population [6, 7]. Another approach is to use retrospectively recalled pre-stroke HRQoL soon after the onset of event. Although both approaches provide useful information about the health burden of stroke survivors and offer practical advantages over prospective measurement of baseline pre-stroke HRQoL, both have limitations. Population norms can be useful reference points for tracking of population health status and for comparison with disease populations. Nonetheless, using population norms as a surrogate of pre-onset HRQoL of disease population may be unrepresentative [8, 9]. On the other hand, while retrospectively recalled pre-stroke HRQoL may provide a more robust estimate since the before and after onset were completed by the same individual with same internal standard of measures, it might suffer from recall bias. Studies that considered pre-stroke HRQoL when quantifying health burden were scarce.

Owing to the limited resources and difficulties in prospectively collecting pre-stroke HRQoL, we quantified the HRQoL loss associated with first-time stroke by using retrospective self-assessment of pre-stroke HRQoL and following up stroke survivors up to 12 month post-stroke in this study. To understand the HRQoL deficits of stroke patients, we evaluated the pre- and post-stroke HRQoL of stroke survivors and compared it with that of the general population. We hypothesized that the HRQoL of stroke survivors at 3 month and 1 year post-stroke were lower than their respective pre-stroke HRQoL, as well as the HRQoL of the general population.

## Methods

### Study population

This study used data collected in the Singapore Stroke Study (S3), which was a one-year prospective, multi-center cohort study on consecutive acute stroke patients and their primary caregivers. The recruitment started in November 2011 and the last follow-up ended in December 2014. Patients who were admitted to inpatient stroke units of five public tertiary hospitals in Singapore, namely Changi General Hospital, Khoo Teck Puat Hospital, National University Hospital, National Neuroscience Institute (NNI) at Tan Tock Seng Hospital, and NNI at Singapore General Hospital were screened for eligibility.

The eligibility criteria for S3 were: 1) Singaporean or permanent resident aged 40 years or above; 2) A recent diagnosis of stroke (stroke symptoms occurring within four weeks prior to enrollment) as confirmed by clinicians and/or supported by neuroimaging; and 3) not globally aphasic. Patients with transient ischemic attack were excluded from the study. In addition to above criteria, in this study, we restricted our study sample to patients with first episode of stroke and able to respond to questionnaire by themselves at each interview time point. (In S3, whenever patients were unable to answer questions on their own, proxy responses were obtained from their caregivers.)

### Recruitment and follow-up

Admitted stroke patients were reviewed by research nurse for eligibility. Eligible subjects were then invited to participate in the study. Full disclosure was provided to the subjects at recruitment before obtaining written informed consent. A structural questionnaire was administered by trained personnel during their inpatient hospital stay. Follow-up home visits were conducted by trained interviewers at 3 and 12 month after discharge. To reduce attrition rate, subjects were reminded one week in advance by mail and phone calls or text messages were made one day before the visit. Multiple attempts (> = 3) were made before categorizing the subjects as lost to follow-up. All procedures performed in this study involving human participants were reviewed and approved by the SingHealth Centralized Institutional Review Board (ref no: 2010/724/A) and National Healthcare Group Domain Specific Review Board (ref no: A/10/690). Written informed consent was obtained from all individual participants included in the study.

### Instruments

The 3-level European Quality of Life Five Dimensions (EQ-5D-3L) [10] was interviewer-administered at recruitment, 3 month and 12 month post-stroke to assess patients’ HRQoL. It has been validated in many countries including Singapore [11–13] with reasonable reliability and responsiveness demonstrated for measuring HRQoL after stroke [14–17]. The instrument consists of five questions, each assessing problems in one of the five dimensions: mobility, self-care, usual activities, pain/discomfort, and anxiety/depression. Each dimension is assigned a level using a 3-point scale: no problems, some or moderate problems, extreme problems. EQ-5D-3L also includes a 100-point visual analogue scale for respondents to assess their general health. However, in the current study, we only focused on responses to the five health dimensions and calculated the EQ index score using a scoring algorithm developed for Singapore [18]. The EQ index score indicates the utility value of a respondent’s health state from the perspective of the general public. It is anchored by 0 (dead) and 1 (full health), with negative scores indicating that a health state is worse than dead. The standard EQ-5D-3L asks respondents to report their health on the day of survey. It was used as it is in the surveys at 3 month and 12 month post-stroke; however, we asked patients to report their health state on a typical day before the onset of current stroke in the survey at recruitment. This health state provided an estimate of the patients’ pre-stroke HRQoL.

Clinical outcome measures performed by interviewers included National Institute of Health Stroke Scale (NIHSS) [19] and Mini-Mental State Examination (MMSE) [20]. The NIHSS is a systematic assessment tool that provides a quantitative measure of stroke-related neurological deficit. The scale is composed of 15 items, including testing of level of consciousness, selected cranial nerves, motor, sensory, cerebellar function, language and inattention. For each item, a score of 0 typically indicates normal function in that particular ability. A score in the range of 1 to 4 indicates level of impairment, with a higher score representing the higher severity level of impairment. The total score is calculated by summing the individual item scores, with the possible maximum score being 42 and minimum score being 0. Based on the total scores, the stroke severity may be categorized as follow: Very Severe > 25 points; Severe 15–24 points; Mild to Moderately Severe 6–14 points; and Mild 1–5 points [21].

The MMSE is widely used in stroke patients to evaluate cognitive impairment and to assess the cognition progression and severity over time [22, 23]. It covers orientation to time and place, immediate recall, attention, delayed recall, language, and construction ability. Each correct answer is scored one point. The total summed score ranges from 0 to 30, with higher scoring indicates better cognitive functioning. In Singapore, the localized MMSE version has been shown to discriminate well between community-living elderly with and without dementia, with a score greater or equal to 24 indicating a normal cognition [24].

### Statistical analysis

Descriptive analysis was performed for socio-demographic, health and clinical variables. The observed change in HRQoL before and after stroke for the stroke patients was determined by comparing the EQ index scores at 3 and 12 month to the pre-stroke EQ index score, respectively, and paired t-test was used to assess the statistical significance of the change between the two time-points. In order to assess the change in health state in the EQ-5D-3L dimensions, we analyzed the change in percentage of patients reporting health problems in each EQ-5D-3L dimension. McNemar test was used to assess the statistical significance of the change in each individual dimension.

Patients’ mean EQ index score pre-stroke and at 3 month and 12 month post-stroke were compared to age, gender and ethnicity adjusted population norms for the EQ index score, estimated using a large sample of the general population in Singapore [25]. Population norm allows comparison of health profiles of patients with specific conditions with those of an average person in the general population with similar demographic characteristics. The normative values can be regarded as anticipated values for a general population sample whose age, gender and ethnicity distributions are same as those of the patient sample [26]. The difference between observed mean EQ index scores and the corresponding normative scores (expected mean) was tested using one-sample test for each time point: pre-stroke, 3 and 12 month post-stroke. To minimize the inflated type 1 error due to multiple comparisons, p < 0.01 was considered significant in this study. All statistical analyses were performed using Stata (version 13.0) [27].

## Results

**Fig 1:**
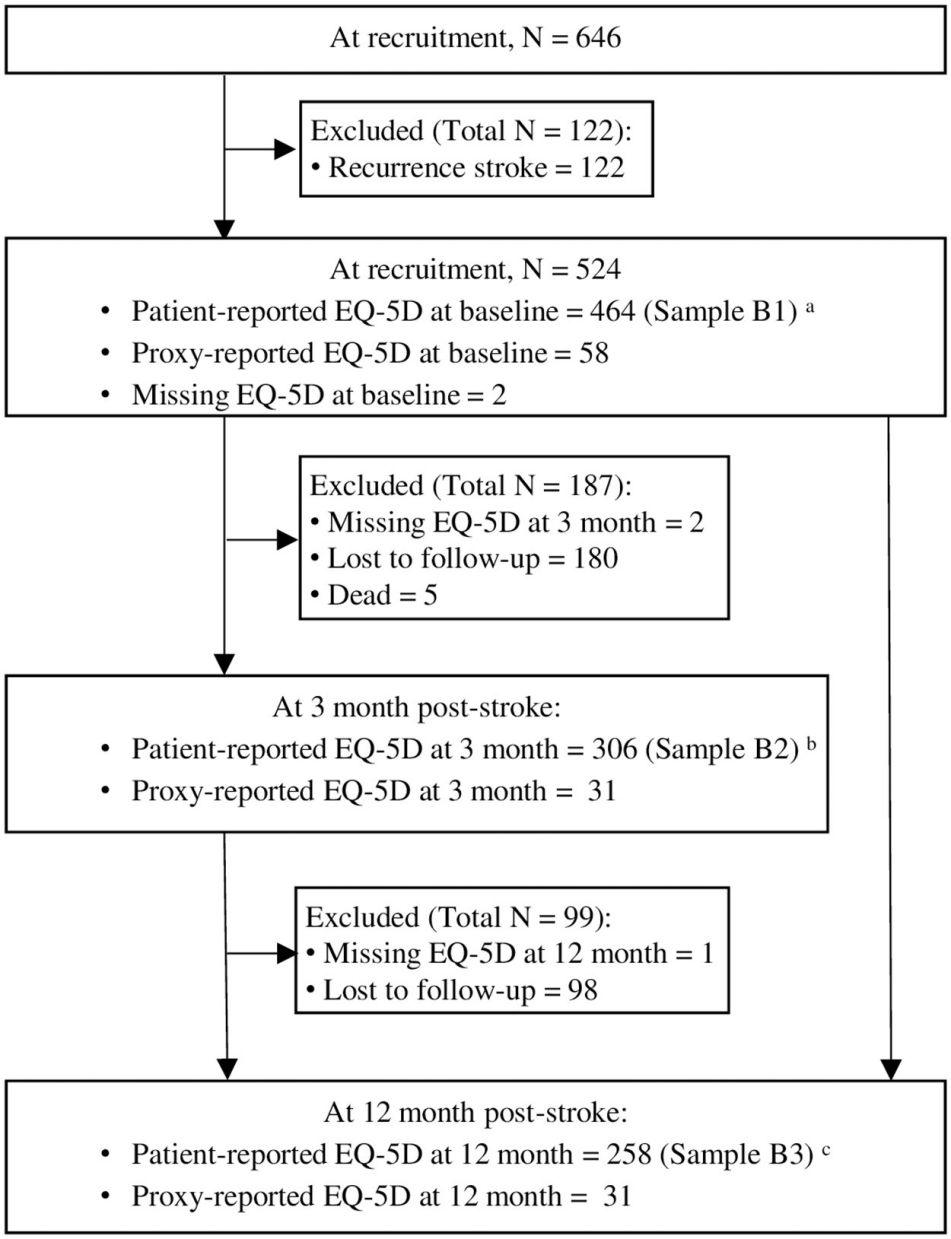
Study flow chart. ^a^ denotes the number of patients included in the cross-sectional comparison with population norms at baseline. ^b^ denotes the number of patients included in the cross-sectional comparison with population norms at 3 month post-stroke. Study sample A1 which assessed the health-related quality of life (HRQoL) change before and 3 months after stroke was a subset of this sample. ^c^ denotes the number of patients included in the cross-sectional comparison with population norms at 12 month post-stroke. Study sample A2 which assessed the HRQoL change before and 12 months after stroke was a subset of this sample. ^a,b,c^ Only patient-reported EQ-5D was included in the analytic samples. Proxy-reported EQ-5D and missing EQ-5D at each time point were excluded from analysis.

A study flow chart is shown in Fig 1. After excluding patients experiencing recurrent stroke, cases answered by caregiver, and cases with missing information in EQ-5D, a total of 464, 306 and 258 patients at baseline (Sample B1), 3 month (Sample B2) and 12 month (Sample B3), respectively, were included for cross-sectional comparison with population norms. At recruitment, the mean age was 61.8 years (standard deviation [SD] = 10.3) and 67.5% of patients were male. Majority of patients were of ethnic Chinese (69.4%), married (71.3%) and of Buddhism/ Taoism faith (48.1%). 52.8% of patients had spouse as their primary caregiver. Ischemic stroke (90.9%) was the main diagnosis. 71.1% of patients reported having hypertension, 68.5% having hyperlipidaemia and 37.1% having diabetes mellitus. Patients were generally having mild stroke (mean NIHSS score = 4.0) with normal cognitive function (mean MMSE > 24). Patients followed-up at 3 month and 12 month had similar characteristics to those surveyed at recruitment except that they were more likely to be married and had a spouse as their primary caregiver. For assessing HRQoL changed before and after stroke, 285 patients were included for assessing change from baseline to 3 month (Sample A1) and 238 patients were included for assessing change from baseline to 12 month (Sample A2). Study samples A1 and A2 were subsets of samples B2 and B3, respectively. The characteristics of the two samples are shown in Table 1.

**Table 1:** Demographic characteristics of study samples at recruitment.

	(A) Study samples for longitudinal comparisons between pre- and post-stroke HRQoL		(B) Study samples for cross-sectional comparisons with population norms		
	(A1) 0–3 month	(A2) 0-12month	(B1) At recruitment	(B2) 3 month post-stroke	(B3) 12 month post-stroke
	N = 285	N = 238	N = 464	N = 306	N = 258
	% or mean (sd)	% or mean (sd)	% or mean (sd)	% or mean (sd)	% or mean (sd)
Age (years)	61.7 (9.9)	60.7 (9.5)	61.8 (10.3)	61.5 (10.1)	60.7 (9.7)
Gender					
Male	70.2	67.6	67.5	69.9	66.3
Female	29.8	32.4	32.5	30.1	33.7
Ethnicity					
Chinese	67.7	68.1	69.4	66.7	66.7
Malay	22.8	21.9	21.3	23.5	23.3
Indian and others	9.5	10.1	9.3	9.8	10.1
Marital status					
Single	9.8	8.4	12.3	9.8	8.5
Married	76.8	77.7	71.3	76.5	76.4
Separated/ Divorced	3.9	5.9	6.0	3.9	5.8
Widowed	9.5	8.0	10.3	9.8	9.3
Primary caregiver					
Spouse	56.9	57.8	52.8	57.9	58.0
Child	22.6	21.5	23.9	22.4	22.6
Sibling	5.0	5.9	6.5	4.9	5.8
Maid/ Others	4.2	5.1	5.0	4.3	4.7
None	11.3	9.7	11.7	10.5	8.9
Religion					
Christianity	11.6	12.2	12.5	11.4	12.4
Buddhism / Taoism	47.4	46.2	48.1	47.1	45.4
Islam	27.0	26.5	24.8	28.1	27.9
Hinduism/ Others	4.6	4.2	5.8	4.3	3.9
No religion	9.5	10.9	8.8	9.2	10.5
Baseline survey mode					
Stroke patient	100.0	100.0	100.0	93.1	92.3
Primary caregiver	0.0	0.0	0.0	6.9	7.7
Subtype of stroke					
Infarct (ischemic)	91.6	91.2	90.9	89.9	89.5
Haemorrhage/ both	8.4	8.8	9.1	10.1	10.5
Comorbidities					
Hypertension	71.6	70.2	71.1	70.9	70.2
Hyperlipidaemia	68.8	66.0	68.5	68.6	66.3
Diabetes mellitus	38.3	39.9	37.1	39.2	41.5
NIHSS	3.9 (3.4)	3.8 (3.5)	4.0 (3.7)	4.0 (3.6)	4.0 (3.8)
MMSE	25.2 (4.3)	25.1 (4.6)	24.5 (4.9)	24.8 (5.0)	24.5 (5.7)

NIHSS, National Institute of Health Stroke Scale; MMSE, Mini-Mental State Examination.

Table 2 shows the observed changed in HRQoL before and after stroke for stroke patients. The mean [99% confidence interval (CI)] EQ index score of patients who were followed up at 3 month fell by 0.25 [0.18; 0.32] points from the pre-stroke baseline (p<0.001); the mean [99% CI] EQ index score of the patients who were followed up at 12 month fell by 0.09 [0.03; 0.15] points from the pre-stroke baseline (p<0.001).

**Table 2:** Comparison of pre- and post-stroke EQ index scores of stroke patients.

	Mean (99% CI)	P value^a^
Pre-stroke to 3 month post-stroke (N = 285)		
Pre-stroke	0.87 (0.84; 0.90)	
3 month post-stroke	0.62 (0.55; 0.68)	
Difference between pre-stroke and 3 month post-stroke	0.25 (0.18; 0.32)	<0.001
Pre-stroke to 12 month post-stroke (N = 238)		
Pre-stroke	0.87 (0.83; 0.90)	
12 month post-stroke	0.78 (0.72; 0.83)	
Difference between pre-stroke and 12 month post-stroke	0.09 (0.03; 0.15)	<0.001

99% CI, 99% confidence interval

^a^ p value of mean difference using paired t-test

**Fig 2:**
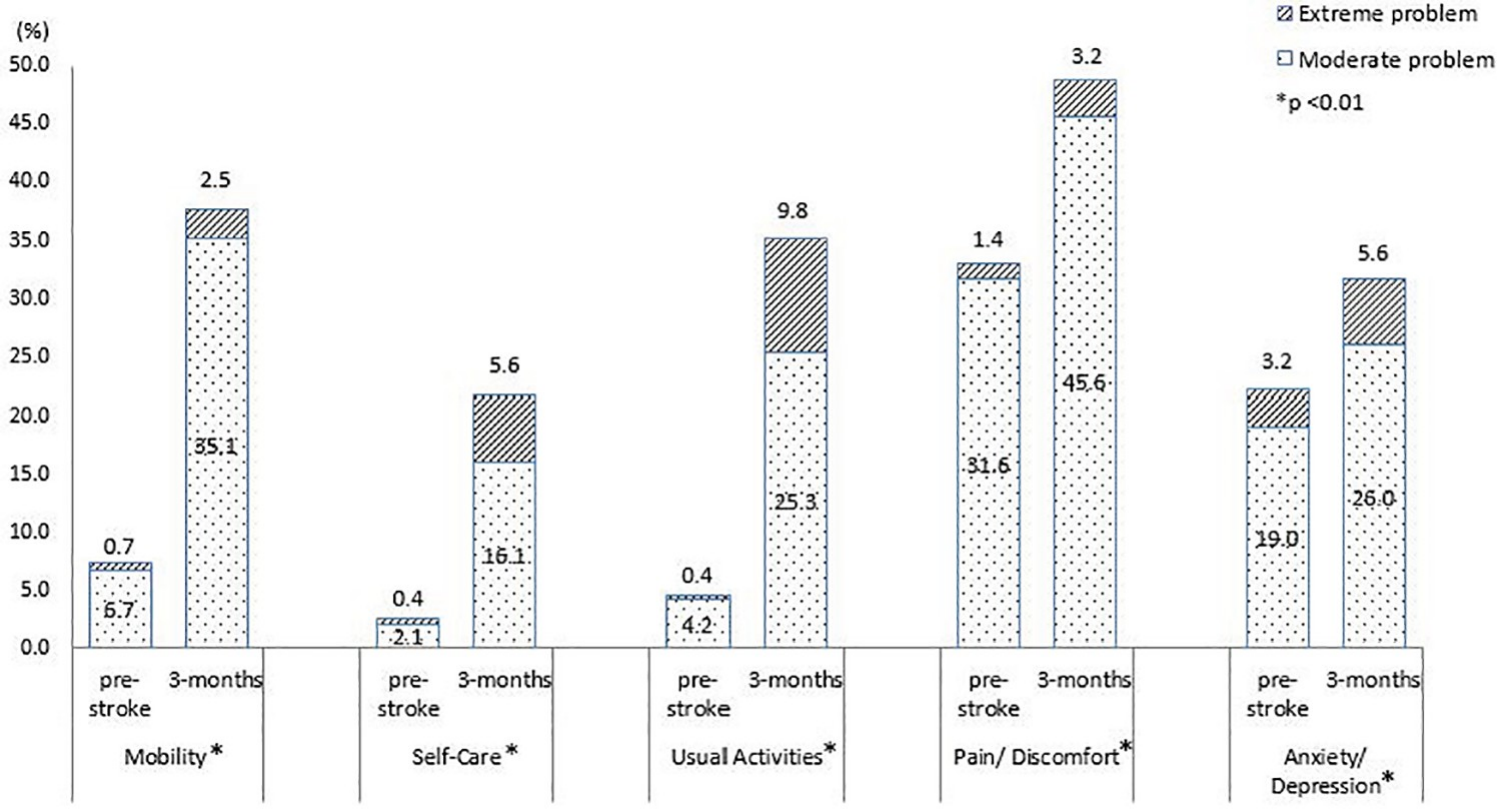
Percentage of stroke survivors reporting problems in individual dimensions before and 3 month after stroke (N = 285).

**Fig 3:**
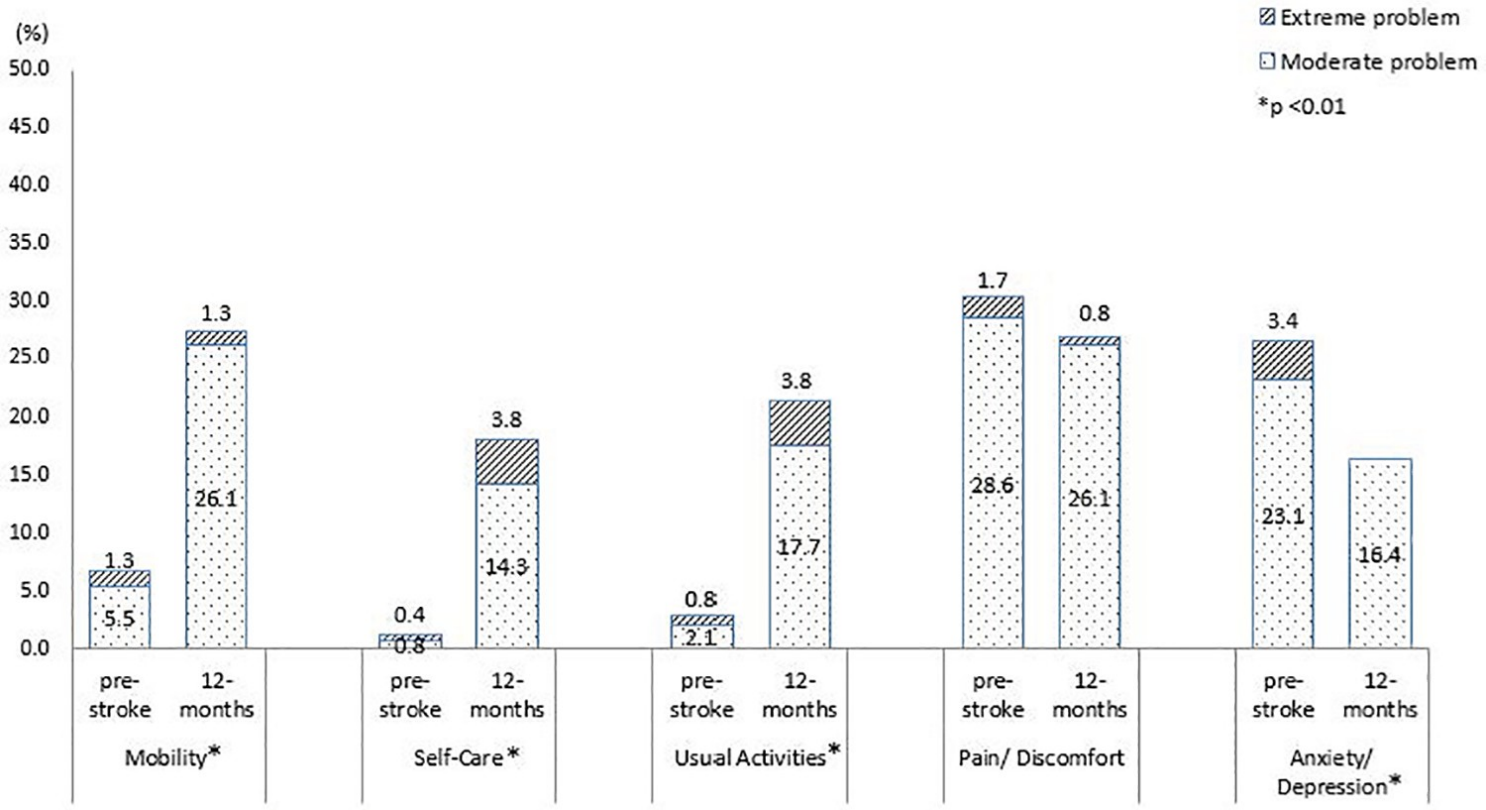
Percentage of stroke survivors reporting problems in individual dimensions before and one year after stroke (N = 238).

The percentage of stroke patients reporting problems in each EQ dimension at pre-stroke and 3 month or 12 month post-stroke was depicted in Figs 2 and 3, respectively. Compared 3 month post-stroke to pre-stroke, the percentage of patients reporting problems at 3 month increased for all dimensions (p<0.01). Among those, mobility (7.4% to 37.6%) and usual activities (4.6% to 35.1%) were the two dimensions with most prominent increase. Pain/discomfort was reported by most patients, at both pre-stroke (33.0%) and 3 month post-stroke (48.8%). For patients who were followed up at one-year post-stroke, there was increase in percentage of patients reporting problems in mobility, self-care and usual activities. A decrease of 3.4% and 10.1% were seen for pain/discomfort and anxiety/depression, respectively, compared to the pre-stroke level. Mobility (27.4%) was the dimension with highest percentage of patients reporting problems at one year post-stroke, followed by pain/ discomfort (26.9%). Significant difference in change in percentage of patients reporting problem pre- and one-year post-stroke was found in all dimensions except pain/discomfort.

Table 3 shows the comparison of the mean EQ index scores of the stroke survivors at pre-stroke, 3 month post-stroke and 12 month post-stroke with their corresponding population norms. Compared with age, gender and ethnicity adjusted population norms, stroke patients had significant lower EQ index scores at all three time points. The expected mean EQ index score for the matched general population was about 0.94 at pre-stroke level, while the observed mean EQ index score for the stroke sample was 0.87 and significantly different from 0.94 (p <0.001). At 3 month post-stroke, the observed mean EQ index score of stroke survivors decreased to 0.61, and increased slightly to 0.76 at 12 month post-stroke, and these scores were significantly different from their expected population mean EQ index score (which was 0.94 for both samples).

**Table 3:** Cross-sectional comparison of EQ index scores with adjusted population norms.

	N	Expected mean^a^	Observed mean^b^ (99% CI)	Difference^c^	P value^d^
Pre-stroke	464	0.94	0.87 (0.84; 0.89)	0.07	<0.001
3 month post-stroke	306	0.94	0.61 (0.55; 0.67)	0.33	<0.001
12 month post-stroke	258	0.94	0.76 (0.71; 0.81)	0.18	<0.001

99% CI, 99% confidence interval

^a^ Expected mean: the age, gender, and ethnicity adjusted mean EQ index scores obtained from published population norm

^b^ Observed mean: the mean EQ index scores of stroke patients in our study

^c^ Difference: Expected mean minus Observed mean

^d^ p value of mean difference using one sample t-test

## Discussion

Comparison of self-reported HRQoL before and after stroke provides the best information on HRQoL loss resulting solely from the onset of stroke. Patients’ perceived pre-stroke HRQoL is an important factor to consider and could certainly influence post-stroke functions over time. Although age-gender standardized population norms was commonly used as reference points to measure the HRQoL loss of disease population, Watson et al. [28] and Wilson et al. [29] demonstrated that retrospective baseline measurement of pre-onset HRQoL is more appropriate than the application of population norms for evaluating post event losses. In this study, we quantified HRQoL loss associated with first-time stroke using recalled pre-stroke HRQoL as the baseline measurement. As shown in our study, the HRQoL of stroke survivors at 3 month was 71% of pre-stroke HRQoL and it was recovered up to 90% of the pre-stroke HRQoL at 12 month.

We found that the increase in health problems from pre-stroke to 3 month was higher than that from pre-stroke to 12 month. This finding was not surprising from the view of recovery course. At 3 month post-stroke, most stroke survivors suffered from certain level of physical dependence and they had limited ability to move around and conducting their usual activities. At one-year post-stroke, patients’ physical functioning was still far less than optimal compared to the pre-stroke status. However, their pain/discomfort and anxiety/depression level were lower than pre-stroke only by 3.4% and 10.1%, respectively, as compared to the pre-stroke level. Perhaps stroke survivors had adapted to the stroke conditions with time and changed their expectations and internal value about their health and life.

Our results showed that HRQoL of the stroke survivors was poorer than that of the Singaporean general population, regardless of whether it was before or after onset of stroke. HRQoL of the stroke survivors was 7.4% lower than that of the general population at before stroke. This deficit could be due to the comorbidities of the stroke. The prevalence of diabetes mellitus (DM), hypertension (HTN) or hyperlipidaemia (HLD) in the study samples (as shown in Table 1) was higher than the age-specific prevalence rate in the general population [30]. The lower HRQoL could also be attributed to undiagnosed mini-stroke or DM/HTN/HLD-related sequelae. At 3 month post-stroke, it was not surprising the reduction in HRQoL was as much as 35.1% compared to the general population, and this figure rose back to 19.1% after one year. The result of 19.1% reduction in HRQoL at one year post-stroke was similar to the findings in studies conducted in South Korea [6] and United States [4]. As compared to population norms, HRQoL of stroke patients reduced by 23% and 21% respectively in South Korea and United States. Moreover, a local study which used EQ-5D-3L of the same scoring algorithm as our study to evaluate HRQoL of patients with first episode psychosis found a reduction of 18% from the population norms [31], suggesting that stroke has a similar impact as psychosis on patients’ HRQoL even on eyear after stroke onset.

The main strength of the study was the data was obtained from a one-year multi-centre longitudinal study which enables comparison of HRQoL before and after stroke within the same study sample. The obtained estimates were subjected to lower risks of confounding effect, thereby providing a more robust estimate of HRQoL loss due to stroke. Although retrospective evaluation of pre-onset health status suffers from recall bias, its usefulness and appropriateness in acute-onset illness have been demonstrated in previous studies, as compared to the application of population norms [28, 29]. The HRQoL instrument used is known for its reliability and validity for the stroke population [15, 32, 33]. It has also been locally validated in four local languages, which satisfied the need of Singapore as a multi-cultural multi-ethnic society [11–13].

This study has some potential limitations. With a prospective follow-up study design, it was inevitable some participants were lost to follow up. Although measures such as home visiting, reminder calls and text messaging, and incentives were taken to retain patients, the loss to follow-up rate was relatively high. However, this was unlikely to have pronounced impact on our findings on HRQoL, as there was no significant difference in the characteristics of the patients who were followed up and those who were not (refer to S1 Table). The pre-stroke HRQoL which was assessed retrospectively might suffer from recall bias. Although recall bias might be a concern given the potential of diminished cognitive functioning, our patients had a mean MMSE score of 24 and above, implying no or mild cognitive decline. Nevertheless, stroke could affect patients’ awareness and insight. To a certain extent, patients’ responses might be affected especially during the baseline assessment. In addition, selection bias might have caused underestimation of poor HRQoL in the study. Patients with severe stroke were unable to self-report their HRQoL. Although proxy responses were available, we excluded those from the current study. Previous studies had reported low level of agreement between responses from proxy and patient [34, 35]. This is especially prominent for the more subjective domains of HRQoL as proxies tend to overrate the severity of a patient’s condition. Therefore, our findings may have limited generalizability to patients with severe stroke. Lastly, EQ-5D is a generic HRQoL measure with only three descriptive levels for each of the five dimensions. The information gained might be limited. Future studies using more sensitive disease-specific instruments are therefore warranted.

## Conclusions

Stroke has substantial impact on HRQoL of Singaporean stroke survivors, especially in the first three month post-stroke. Compared to the general population, stroke survivors have lower HRQoL even before stroke onset. This difference should be taken into account when quantifying health burden of stroke or setting the target of rehabilitation for stroke survivors.

## Supporting information

S1 Table Baseline characteristics of S3 study participants: Follow-up and loss to follow-up comparison at 3 month and 12 month. (PDF)
