# Supplementary material for: Health-related quality of life loss associated with first-time stroke
Source: PLoS One. 2019 Jan 28;14(1):e0211493. doi: 10.1371/journal.pone.0211493 (PMC6349359; doi:10.1371/journal.pone.0211493)
Supplement: S1 Table — (PDF) [file pone.0211493.s001.pdf]

S1 Table. Baseline characteristics of S3 study participants: follow-up and loss to follow-up comparison at 3 month and 12 month.

	Follow-up at 3 months (N = 419)	Loss to follow-up at 3 months (N = 242)		Follow-up at 12 months (N =361)	Loss to follow-up at 12 months (N = 300)	
	N (%)	N (%)	p value	N (%)	N (%)	p value
**Age**, mean (sd)	62.6 (10.8)	63.3 (11.8)	0.499	62.6 (10.8)	63.2 (11.6)	0.369
**Gender**			0.949			0.399
Male	276 (65.9)	160 (66.1)		233 (64.5)	203 (67.7)	
Female	143 (34.1)	82 (33.9)		128 (35.5)	97 (32.3)	
**Ethnicity**			0.048			0.143
Chinese	269 (64.2)	177 (73.1)		233 (64.5)	213 (71.0)	
Malay	109 (26.0)	44 (18.2)		94 (26.0)	59 (19.7)	
Indian and others	41 (9.8)	21 (8.7)		34 (9.4)	28 (9.3)	
**Marital status**			0.001			0.015
Single	40 (9.6)	37 (15.3)		32 (8.9)	45 (15.0)	
Married	305 (72.8)	147 (60.7)		265 (73.4)	187 (62.3)	
Separated/ Divorced	17 (4.1)	24 (9.9)		21 (5.8)	20 (6.7)	
Widowed	57 (13.6)	34 (14.1)		43 (11.9)	48 (16.0)	
**Primary caregiver**			0.227			0.102
Spouse	219 (52.5)	107 (44.6)		193 (53.6)	133 (44.8)	
Child	112 (26.9)	68 (28.3)		92 (25.6)	88 (29.6)	
Sibling	21 (5.0)	20 (8.3)		22 (6.1)	19 (6.4)	
Maid/ others	22 (5.3)	16 (6.7)		22 (6.1)	16 (5.4)	
None	43 (10.3)	29 (12.1)		31 (8.6)	41 (13.8)	
**Religion**			0.020			0.024
Christianity	48 (11.5)	36 (14.9)		43 (11.9)	41 (13.7)	
Buddhism / Taoism	188 (44.9)	123 (50.8)		161 (44.6)	150 (50.0)	
Islam	125 (29.8)	47 (19.4)		107 (29.7)	65 (21.7)	

Hinduism/ Others	18 (4.3)	17 (7.0)		13 (3.6)	22 (7.3)	
No religion	40 (9.6)	19 (7.9)		37 (10.3)	22 (7.3)	
**Restructured hospital**			0.130			0.074
NNI @ TTSH	109 (26.0)	50 (20.7)		93 (25.8)	66 (22.0)	
NNI @ SGH	78 (18.6)	33 (13.6)		55 (15.2)	56 (18.7)	
NUH	16 (3.8)	10 (4.1)		14 (3.9)	12 (4.0)	
KTPH	92 (22.0)	60 (24.8)		71 (19.7)	81 (27.0)	
CGH	124 (29.6)	89 (36.8)		128 (35.5)	85 (28.3)	
**Ward class**			0.638			0.518
B2 and higher	214 (51.1)	119 (49.2)		186 (51.5)	147 (49.0)	
C	205 (48.9)	123 (50.8)		175 (48.5)	153 (51.0)	
**Survey mode at baseline**			0.960			0.540
Stroke patient	372 (89.0)	213 (89.1)		319 (88.4)	266 (89.9)	
Primary caregiver	46 (11.0)	26 (10.9)		42 (11.6)	30 (10.1)	
**Subtype of stroke**			0.902			0.676
Infarct (ischemic)	368 (87.8)	210 (87.5)		314 (87.2)	264 (88.3)	
Haemorrhage (bleed)/ both	51 (12.2)	30 (12.5)		46 (12.8)	35 (11.7)	
**Episode of stroke**			0.816			0.706
First stroke	339 (80.9)	194 (80.2)		293 (81.2)	240 (80.0)	
Recurrent (2nd or subsequent stroke)	80 (19.1)	48 (19.8)		68 (18.8)	60 (20.0)	
**Hypertension**			0.499			0.169
Yes	308 (73.5)	172 (71.1)		270 (74.8)	210 (70.0)	
No	111 (26.5)	70 (28.9)		91 (25.2)	90 (30.0)	
**Hyperlipidaemia**			0.834			0.772
Yes	301 (71.8)	172 (71.1)		260 (72.0)	213 (71.0)	
No	118 (28.2)	70 (28.9)		101 (28.0)	87 (29.0)	
**Diabetes Mellitus**			0.479			0.100
Yes	178 (42.5)	96 (39.7)		160 (44.3)	114 (38.0)	

No	241 (57.5)	146 (60.3)		201 (55.7)	186 (62.0)	
**pre-stroke EQ index scores,** mean (sd)	0.82 (0.27)	0.81 (0.30)	0.792	0.82 (0.28)	0.82 (0.28)	0.794
**NIHSS**, mean (sd)	4.87 (4.77)	4.76 (4.72)	0.697	4.72 (4.55)	4.96 (4.99)	0.712
**MMSE**, mean (sd)	23.42 (6.33)	23.01 (6.38)	0.314	23.32 (6.48)	23.22 (6.18)	0.533

NIHSS, National Institute of Health Stroke Scale; MMSE, Mini-Mental State Examination
